# Supplementary material for: Perceptions of a gender-neutral approach to human papillomavirus (HPV) vaccination in Cameroon: a qualitative study
Source: BMC Public Health. 2026 Feb 23;26:1043. doi: 10.1186/s12889-026-26730-9 (PMC13037256; doi:10.1186/s12889-026-26730-9)
Supplement: Supplementary file 1 — Supplementary Material 1. [file 12889_2026_26730_MOESM1_ESM.pdf]

Interview Guide (Central Coordination EPI)

Introduction

Hello, my name is .... I am conducting this interview on behalf of the EPI, in collaboration with CHAI, for the study on the evaluation of the effect of gender-neutral vaccination on the uptake of HPV vaccination in nine-year-old girls in Cameroon. This study aims to determine the effect of Gender-Neutral Vaccination (GNV) and other HPV-related interventions on the uptake and acceptance of HPV vaccination. Rest assured that the information obtained during this interview will not have any detrimental administrative consequences. We will ensure that all the answers you give to our questions remain completely anonymous. However, with your permission, the interview will be recorded to ensure that your ideas and proposals are true, and that none of them are forgotten.

A. Context

1. How long have you been in this position?
2. Can you tell us about your role and responsibilities within the program?
3. How has HPV vaccination in nine-year-old girls evolved since its introduction into the routine immunization program in Cameroon?
4. What resistance/constraints have you encountered since the introduction of HPV vaccination?
   Prompt: Demand more details on each constraint and find out if health workers are also among those who are fuelling resistance.
5. What strategies have been used at the central level to address these constraints to improve uptake of the HPV vaccine?
   Prompt: Possibly emphasize the strategies used with regard to the health workforce
6. To what extent has offering a single dose affected the uptake of the HPV vaccine?

B. Introduction of the gender-neutral strategy

1. What can you tell us about the gender-neutral strategy?
2. Why did you decide to adopt it?
3. What are the key factors considered in the adoption and implementation of the gender-neutral vaccination (GNV) approach for the HPV vaccine in Cameroon?

C. Effect of GNV on HPV vaccine uptake

1. From your perspective, how has the gender-neutral vaccination approach affected the uptake of the HPV vaccine among 9-year-old girls in Cameroon?
2. What are the specific or major changes that you have perceived following the adoption of the gender-neutral approach?
   Prompt: Emphasize each specific change

3. In your opinion, what can be the contribution of the gender-neutral approach to HPV vaccination to gender equality, health equity and social inclusion?

D. Implementation of the gender-neutral vaccination approach by districts

1. What are the main challenges or obstacles that the different districts have faced in implementing the gender-neutral vaccination approach for the HPV vaccine?

Prompt

- *Could you identify specific challenges related to human resources, logistics, finance, communication, or service delivery that have hindered the implementation for the success of the gender-neutral approach to immunization?*

2. What strategies or different interventions do you think have been implemented to overcome these barriers and improve the success of the GNV approach?

E. Linking Periodic Intensification of Routine Immunization and Community Dialogue Strategies and to the Gender-Neutral Approach

1. What has been the impact of **Periodic Intensification of Routine Immunization** and community dialogues (CDs) conducted in specific health a reason HPV vaccine uptake?

Prompt

- *What are the most illustrative examples?*

2. What are the challenges encountered in the implementation of these activities that could affect the uptake of the HPV vaccine?
3. How did you overcome them?
4. Can you share with us the specific views on the impact of these activities on the perceptions and decisions of different stakeholders regarding the vaccine in different regions and districts?
5. Are there any activities other than those mentioned above that have influenced the uptake of HPV vaccines? Please elaborate

F. **Collaboration between the EPI and other stakeholders in the implementation of the gender-neutral approach**

1. How do you collaborate with other stakeholders in the implementation of the GNV approach?

Prompt: Emphasize collaboration with schools, CSOs, and other stakeholders

2. What specific role(s) did each stakeholder play in facilitating the implementation of the GNV approach and the uptake of HPV vaccination in 9-year-old girls?
3. What is the importance of this collaboration? Justify
4. What are the challenges of this collaboration?
5. How can they be overcome?
   Prompt: contribution from the informant and other stakeholders

G. Best practices and recommendations

1. In your opinion, what are the best practices or effective strategies in the implementation of the GNV approach for HPV vaccine?
   Prompt *Highlight specific examples of successful interventions or initiatives that have had a positive influence on HPV vaccine uptake*
2. In your opinion, what are the best practices or effective strategies in the implementation of the GNV strategy in combination with the PIRI and CD approaches for HPV vaccine?
   Prompt: Highlight specific examples of successful interventions or initiatives that have had a positive influence on HPV vaccine acceptance
3. What specific areas or aspects do you think need to be prioritized or addressed to ensure the long-term success of the GNV approach for HPV vaccine?
4. Are there any other ideas or recommendations you would like to share regarding the GNV approach?
5. In general, what other recommendations could you make to further increase HPV vaccination coverage among 9-year-old girls in Cameroon
6. What do you think about the long-term implementation of the gender-neutral strategy?

DO YOU HAVE ANYTHING ELSE TO ADD?

INTERVIEW GUIDE (for DISTRICT MEDICAL OFFICERS)

Introduction

Hello, my name is .... I am conducting this interview on behalf of the EPI, in collaboration with CHAI, for the study on the evaluation of the effect of gender-neutral vaccination on the uptake of HPV vaccination in nine-year-old girls in Cameroon. This study aims to determine the effect of Gender-Neutral Vaccination (GNV) and other HPV-related interventions on the uptake and acceptance of HPV vaccination. Rest assured that the information obtained during this interview will not have any detrimental administrative consequences. We will ensure that all the answers you give to our questions remain completely anonymous. However, with your permission, the interview will be recorded to ensure that your ideas and proposals are true, and that none of them are forgotten.

A. Background

1. How long have you been in this role?
2. Can you tell us about your role and responsibilities within your district?
3. Can you tell us about the HPV vaccine?
4. How has HPV vaccination for nine-year-old girls evolved since it was introduced into the routine immunization program in your district?
5. What resistance/constraints have you encountered since the introduction of HPV vaccination?
   Prompt(s): Demand more details on each constraint, find out if health workers are also contributing to the resistance.
6. What strategies have been used at the district level to address these constraints to improve the uptake of the HPV vaccine?
   Prompt(s): Possibly emphasize the strategies used with regard to the health workforce
7. What do you think are the specific factors that influence the uptake of the HPV vaccine in your district?
   Prompt(s): Request more details about each factor

B. Introduction of the gender-neutral strategy

1. What can you tell us about the gender-neutral strategy?
2. What do you think justifies the adoption of the gender-neutral strategy?
3. What are the main factors considered in the implementation of the gender-neutral vaccination (GNV) strategy for HPV vaccine in your district?

C. Effect of GNV on HPV vaccine uptake

1. From your perspective, how has the gender-neutral vaccination approach impacted the uptake of the HPV vaccine among 9-year-old girls in your district?
2. What are the specific or major changes that you have perceived following the adoption of the gender-neutral strategy?

   Prompt(s): Emphasize each specific change
3. In your opinion, what can be the contribution of the gender-neutral approach to HPV vaccination to gender equality, health equity and social inclusion?

D. Implementation of the gender-neutral vaccination approach by districts

1. What are the main challenges or obstacles your district has faced in implementing the gender-neutral vaccination strategy to the HPV vaccine?
2. What are the main challenges or barriers that health areas and facilities in your district have faced in implementing the gender-neutral vaccination approach for the HPV vaccine?

   Prompt(s): specific challenges related to human resources, logistics, finance, communication, or service delivery?

3. What strategies or different interventions do you think have been implemented to overcome these barriers and improve the success of the GNV approach?

E. Combining community dialogue strategies and periodic intensification of routine immunization (PIRI), with a gender-neutral approach (DISTRICTS THAT HAVE IMPLEMENTED PIRI AND CD)

1. What have been the impacts of periodic intensification of routine immunization (PIRI), and community dialogues (CDs) conducted in specific health areas in HPV vaccine uptake?

   Prompt(s): Name the most illustrative examples
2. What are the challenges encountered in the implementation of these activities that could affect the uptake of the HPV vaccine?
3. How did you overcome them?
4. Can you share with us the impact of these activities on the perceptions and decisions of different stakeholders (community/religious leaders, parents, schools, health workers, etc.) regarding the vaccine in this district?
5. Are there any activities other than those mentioned above that have influenced the uptake of HPV vaccines in this district? Please give details

F. Combining strategies for community dialogues and periodic intensification of routine immunization (PIRI), with a gender-neutral approach (DISTRICTS THAT HAVE NOT IMPLEMENTED PIRI AND CD)

1. What can you tell us about the PIRI and CD

   Prompt: insist on PIRI and CD related to HPV

2. Do you think that combining these activities with the gender-neutral approach in your district can have a significant effect on HPV vaccine uptake? Why?

G. Collaboration between the District and other stakeholders in the implementation of the gender-neutral approach

1. How do you collaborate with other stakeholders in the implementation of the GNV approach?

   Prompt(s): Emphasize collaboration with schools, CSOs, and other stakeholders

2. What specific role(s) did each stakeholder play in facilitating the implementation of the GNV approach and the uptake of HPV vaccination in 9-year-old girls?
3. What is the importance of this collaboration? Justify
4. What are the challenges of this collaboration?
5. How can they be overcome?

H. Best Practices and Recommendations

1. In your opinion, what are the best practices or effective strategies in the implementation of the GNV approach for the HPV vaccine in your district?

   Prompt(s): Highlight specific examples of successful interventions or initiatives that have had a positive influence on HPV vaccine uptake

2. In your opinion, what are the best practices or effective strategies in the implementation of the GNV strategy in combination with the PIRI and CD approaches for HPV vaccine? (only for districts that have experimented with PIRI and CD)

   Prompt: Highlight specific examples of successful interventions or initiatives that have had a positive influence on HPV vaccine acceptance

3. What specific areas or aspects do you think need to be prioritized or addressed to ensure the long-term success of the GNV approach for the HPV vaccine?
4. Are there any other ideas or recommendations you would like to share regarding the GNV approach?
5. In general, what other recommendations could you make to further increase HPV vaccination coverage among 9-year-old girls in Cameroon?
6. What do you think about the long-term implementation of the gender-neutral strategy?

DO YOU HAVE ANYTHING ELSE TO ADD?

INTERVIEW GUIDE (FOR HEALTHCARE PERSONNEL/EPI FOCAL POINTS)

Introduction

Hello, my name is .... I am conducting this interview on behalf of the EPI, in collaboration with CHAI, for the study on the evaluation of the effect of gender-neutral vaccination on the uptake of HPV vaccination in nine-year-old girls in Cameroon. This study aims to determine the effect of Gender-Neutral Vaccination (GNV) and other HPV-related interventions on the uptake and acceptance of HPV vaccination. Rest assured that the information obtained during this interview will not have any detrimental administrative consequences. We will ensure that all the answers you give to our questions remain completely anonymous. However, with your permission, the interview will be recorded to ensure that your ideas and proposals are true, and that none of them are forgotten.

A. Views and experiences with HPV vaccination

1. How long have you been in this role?
2. Can you tell us about your role and responsibilities within this Health Facility (HF) regarding vaccination?
3. What knowledge/information do you have about the HPV vaccine?
4. What is your opinion on the HPV vaccine?

   Prompt (s): Benefit or not for the girl and justify; Possible recommendation to a patient.
5. Can you describe your experience of HPV vaccination in your HF?

   Prompt(s): Trends, reasons for changes
6. What are the main resistances/constraints you have encountered since the introduction of HPV vaccination?

   Prompt(s): Demand more details on each constraint, find out if health workers are also among those who are fuelling resistance.
7. What strategies have been used at HF to address these constraints to improve HPV vaccine uptake?

   Prompt(s): Possibly emphasize the strategies used regarding the health workforce
8. What do you think are the specific factors that influence the uptake rate of the HPV vaccine in your HF?

   Prompt(s): Request more details about each factor

B. Introduction of the gender-neutral strategy

1. What can you tell us about the gender-neutral strategy?
2. What do you think justifies the adoption of the non-gendered strategy?

3. What are the key factors considered in the implementation of the gender-neutral vaccination (GNV) approach for HPV vaccine in your HF?

C. Effect of GNV on HPV vaccine uptake

1. From your perspective, how has the gender-neutral vaccination approach affected HPV vaccine uptake in 9-year-old girls in your HF?
2. What are the specific or major changes that you have perceived following the adoption of the gender-neutral approach?

Prompt(s): Emphasize each specific change

3. In your opinion, what can be the contribution of the gender-neutral approach to HPV vaccination to gender equality, health equity and social inclusion?

D. Implementation of the gender-neutral vaccination approach by HFs

1. What are the main challenges or obstacles your **HF** has faced in implementing the gender-neutral vaccination approach for HPV vaccine?

Prompt(s): specific challenges related to human resources, logistics, finance, communication, or service delivery

2. What strategies or different interventions do you think have implemented to overcome these barriers and improve the success of the GNV approach?

E. Linking Community Dialogue Strategies and Periodic Intensification of Routine Immunization (PIRIs) to the Gender-Neutral Approach (HF HAVING IMPLEMENTED PIRI AND CD)

1. What have been the impacts of the Periodic Intensification of Routine Immunization (PIRI) and Community Dialogues (CD) conducted in your specific health area in the uptake of the HPV vaccine?

Prompt(s): Name the most illustrative examples (impacts)

2. What are the challenges encountered in the implementation of these activities that could affect the uptake of the HPV vaccine?

Recovery: Challenges related to the appropriation of these activities by the health workforce

3. How did you overcome them?

Prompt(s): Possibly focus on health personnel

4. What is the impact of these activities on the perceptions and decisions of different stakeholders (community/religious leaders, parents, schools, health workers, etc.) regarding the vaccine in your health area?
5. Are there any activities other than those mentioned above that have influenced the uptake of HPV vaccines? Please elaborate

F. Combining community dialogues and PIRIs with a gender-neutral approach (HF NOT HAVING IMPLEMENTED PIRI AND CD)

1. What can you tell us about the PIRIs and CDs *Prompt: insist on the PIRI and CD linked to HPV*
2. Do you think that combining these activities with the gender-neutral approach in your health area can have a significant effect on HPV vaccine uptake? Why?

G. Collaboration between HF and other stakeholders in the implementation of the gender-neutral approach

1. How do you collaborate with other stakeholders in the implementation of the GNV approach?

Prompt(s): Emphasize collaboration with schools, CSOs, and other stakeholders

2. What specific role(s) did each stakeholder play in facilitating the implementation of the GNV approach and the uptake of HPV vaccination in 9-year-old girls?
3. What is the importance of this collaboration? Justify
4. What are the challenges of this collaboration?
5. How can they be overcome?

H. Best Practices and Recommendations

1. In your opinion, what are the best practices or effective strategies in the implementation of the GNV approach for HPV vaccine?
    1. *Prompt(s)(s): Highlight specific examples of successful interventions or initiatives that have had a positive influence on HPV vaccine uptake*
2. In your opinion, what are the best practices or effective strategies in the implementation of the GNV strategy in combination with the PIRI and CD approaches for HPV vaccine? (only for HF who have experimented with PIRI and CD)
    1. ***Prompt: Highlight specific examples of successful interventions or initiatives that have had a positive influence on HPV vaccine acceptance***
3. What specific areas or aspects do you think need to be prioritized or addressed to ensure the long-term success of the GNV approach for HPV vaccine?
4. Are there any other ideas or recommendations you would like to share regarding the GNV approach?
5. In general, what other recommendations could you make to further increase HPV vaccination coverage among 9-year-old girls in Cameroon?
6. What do you think about the long-term implementation of the gender-neutral strategy?

DO YOU HAVE ANYTHING ELSE TO ADD?

INTERVIEW GUIDE (for Ministry of Basic Education Representative)

Introduction

Hello, my name is .... I am conducting this interview on behalf of the EPI, in collaboration with CHAI, for the study on the evaluation of the effect of gender-neutral vaccination on the uptake of HPV vaccination in nine-year-old girls in Cameroon. This study aims to determine the effect of Gender-Neutral Vaccination (GNV) and other HPV-related interventions on the uptake and acceptance of HPV vaccination. Rest assured that the information obtained during this interview will not have any detrimental administrative consequences. We will ensure that all the answers you give to our questions remain completely anonymous. However, with your permission, the interview will be recorded to ensure that your ideas and proposals are true, and that none of them are forgotten.

A. General perceptions and participation in HPV vaccination

1. How long have you been in this role?
2. Can you tell us about your role and responsibilities within the Ministry of Basic Education?
3. What knowledge/information do you have about the HPV vaccine?
4. What is your opinion on the HPV vaccine?
   Prompt: Is it beneficial or not for the girl child? justify
5. What is the role of the Ministry and its schools in promoting and facilitating HPV vaccination among students?
6. How do you (the Ministry and its schools) collaborate with the Ministry of Health on HPV vaccination initiatives?
7. What are the challenges of this collaboration?
8. What recommendations can you make?

B. Knowledge and perceptions of the GNV approach

1. What knowledge do you have about the gender-neutral vaccination (GNV) approach currently used for the HPV vaccine in Cameroon?
   Prompt: Possible influence on your perception of the vaccine
2. What influence has the gender-neutral approach had on parental/community acceptance of the HPV vaccine?
3. In the context of the implementation of the GNV approach, what are the potential challenges that could limit its effectiveness in improving the uptake of the HPV vaccine?
4. In your opinion, what could facilitate the success of the GNV approach?
5. How do you assess this long-term approach in relation to improving the uptake of the HPV vaccine?

C. HPV Vaccine Uptake: Barriers and Facilitators

1. In your opinion, what are the main barriers to the uptake of the HPV vaccine among young girls in Cameroon?
   Prompt: Emphasize school-related barriers

2. How can they be overcome?
Prompt: Emphasize strategies to be deployed in schools

3. What are the main factors that could improve the adoption and uptake of the HPV vaccine among young girls in Cameroon?
Prompt: Emphasize school-related factors

D. Best Practices and Recommendations

1. In your opinion, what are the best practices or effective strategies in the implementation of the GNV approach for the HPV vaccine?
2. Based on your experience, what are the recommendations you can make to improve the uptake of the HPV vaccine in general, and in particular, the implementation of the GNV approach?

Is there any other information or idea you would like to share regarding the influence of the GNV approach on HPV uptake? Or HPV vaccination as a whole?

INTERVIEW GUIDE (for PARENTS with a female child - between 9 and 13 years old)

Introduction

Hello, my name is .... I am conducting this interview on behalf of the EPI, in collaboration with CHAI, for the study on the evaluation of the effect of gender-neutral vaccination on the uptake of HPV vaccination in nine-year-old girls in Cameroon. This study aims to determine the effect of Gender-Neutral Vaccination (GNV) and other HPV-related interventions on the uptake and acceptance of HPV vaccination. Rest assured that the information obtained during this interview will not have any detrimental administrative consequences. We will ensure that all the answers you give to our questions remain completely anonymous. However, with your permission, the interview will be recorded to ensure that your ideas and proposals are true, and that none of them are forgotten.

A. Knowledge and Perspectives on HPV and HPV Vaccination

1. What do you know about cervical cancer?
2. What do you think is causing it?
   Prompt(s): Explore their possible knowledge of HPV
3. Are you aware of the HPV vaccine offered to children in your community?
   Prompt: Emphasize on the potential concerns about HPV vaccine
4. How did you hear about the HPV vaccine?
5. Has your child received the HPV vaccine? (if possible, ask for the vaccination card)
   Prompt(s): Indicate the specific factors that led to your favourable or unfavourable decision. (e.g., referral of a health care provider, community awareness campaigns, religious beliefs)
6. What do you think other members of the community think about HPV vaccination? (Only for parents of children who have received the HPV vaccine)

B. GNV approach: for the vaccinated (parents who have vaccinated their children)

1. Did you know that the HPV vaccine is currently being administered to boys and girls in Cameroon?
   Prompt(s): Find out how he/she found out
2. How did the HPV vaccine for girls and boys affect your decision to vaccinate your child?
3. Do you think the GNV approach could encourage more parents to vaccinate their daughters compared to a girls-only program? Why or why not?

C- GNV approach: for the unvaccinated (parents who have not vaccinated their children)

1. Did you know that the HPV vaccine is currently being administered to boys and girls in Cameroon?
   Prompt(s): Find out how he found out.
2. Would you be more likely to consider getting your daughter vaccinated now, given the current inclusion of boys in the vaccination program, unlike back then? Why?

3. Do you think the GNV approach could encourage more parents to get their daughters vaccinated? Why?

D. Community-based interventions related to HPV

1. Have you participated in any community education or awareness-raising activities related to HPV vaccination? Why?
   Prompt(s): If yes, emphasize the information received about the HPV vaccine.
2. If so, how have these activities influenced your perception of the vaccine?

Prompt(s):- Favourable or unfavourable opinion; Willingness to vaccinate or not

3. Do you think such activities could encourage more parents to get their children vaccinated? Why?

E. Barriers and Facilitators

1. In your experience, what are the main reasons why other parents in your community choose not to have their children vaccinated against HPV?
2. What factors do you think are encouraging other parents in your community to get their children vaccinated against HPV?

E. Recommendations

1. What recommendations can you make to improve the uptake of the HPV vaccine in the community? prompt: return to GNV on the one hand and the PIRI, CD and GNV association on the other hand

2. What other things would you like to share with us about your experience and perspective on HPV vaccination and the GNV approach?

DO YOU HAVE ANYTHING ELSE TO ADD?

INTERVIEW GUIDE (for religious leaders)

Hello, my name is .... I am conducting this interview on behalf of the EPI, in collaboration with CHAI, for the study on the evaluation of the effect of gender-neutral vaccination on the uptake of HPV vaccination in nine-year-old girls in Cameroon. This study aims to determine the effect of Gender-Neutral Vaccination (GNV) and other HPV-related interventions on the uptake and acceptance of HPV vaccination. Rest assured that the information obtained during this interview will not have any detrimental administrative consequences. We will ensure that all the answers you give to our questions remain completely anonymous. However, with your permission, the interview will be recorded to ensure that your ideas and proposals are true, and that none of them are forgotten.

A. Views and experiences of HPV vaccination

1. How long have you been in this role?
2. Can you tell us about your role and responsibilities as religious leader within this community in relation to vaccination?
3. What knowledge/information do you have about the HPV vaccine?
4. What is your opinion on the HPV vaccine?

   Prompt(s): Benefit (or not) for the girl child and justify

5. What does your religious assembly think of the HPV vaccine?

   Prompt(s): Benefit (or not) for the girl child and justify

6. Has your religious assembly participated in activities related to HPV vaccination for young girls? *If so, what is your role in these activities? If not, why didn't she participate?*

7. How do you collaborate with other stakeholders in the implementation of HPV vaccination activities?
   Prompt(s): Emphasize collaboration with HFs, schools, CSOs, and other stakeholders

B. Knowledge and perceptions of the GNV approach

1. Do you know that the HPV vaccine is currently being administered to boys and girls in Cameroon?
   Prompt(s): Find out how he found out
2. What influence did the administration of the HPV vaccine to girls and boys have on the decision of your congregation members to have their children vaccinated?
3. Given the implementation of the GNV approach, what are the potential challenges that could hinder its effectiveness in improving the uptake of the HPV vaccine in this community?
4. Are there any specific factors that could facilitate the success of the GNV approach in this congregation? Why?

C. Linking Community Dialogue Strategies and Periodic Intensification of Routine Immunisation to the Gender-Neutral Approach (COMMUNITY IMPLEMENTING PIRIs AND CD)

1. What have been the impacts of the Periodic Intensification of Routine Immunization (PIRIs) and Community Dialogues (CDs) conducted in your community for HPV vaccine uptake?

Prompt(s): Name the most illustrative examples

2. What are the key challenges encountered in the implementation of these activities that could affect the uptake of the HPV vaccine?

   Prompt(s): Challenges related to the ownership of these activities by health personnel

3. How were they u overcome?
4. What is the impact of these activities on the perceptions and decisions of different stakeholders (traditional leaders, parents, schools, etc.) regarding the vaccine in your community?
5. Are there any activities other than those mentioned above that have influenced the uptake of HPV vaccines? Please elaborate

D. Linking Community Dialogue Strategies and Periodic Intensification of Routine Immunisation to the Gender-Neutral Approach (COMMUNITY NOT IMPLEMENTING PIRIs AND CD)

1. What can you tell us about PIRIs and CD in your community?
   Prompt: insist on PIRI and CD related to HPV

2. Do you think that the combination of these activities with the gender-neutral approach in your health area can have a significant effect on the uptake of the HPV vaccine? Why?

E. Barriers and Facilitators

1. Based on your experience, what are the main reasons why some members of your congregation choose not to have their children vaccinated against HPV?
2. What factors do you think have encouraged other members of your congregation to have their children vaccinated against HPV?

   Prompt(s): Provide examples of successful strategies or initiatives observed.

F. Recommendations

1. What recommendations can you make to improve the uptake of the HPV vaccine in the community?
2. What other things would you like to share with us about your experience and perspective on HPV vaccination and the GNV approach?

DO YOU HAVE ANYTHING ELSE TO ADD?

INTERVIEW GUIDE (for traditional leaders)

Introduction

Hello, my name is .... I am conducting this interview on behalf of the EPI, in collaboration with CHAI, for the study on the evaluation of the effect of gender-neutral vaccination on the uptake of HPV vaccination in nine-year-old girls in Cameroon. This study aims to determine the effect of Gender-Neutral Vaccination (GNV) and other HPV-related interventions on the uptake and acceptance of HPV vaccination. Rest assured that the information obtained during this interview will not have any detrimental administrative consequences. We will ensure that all the answers you give to our questions remain completely anonymous. However, with your permission, the interview will be recorded to ensure that your ideas and proposals are true, and that none of them are forgotten.

A. Views and experiences of HPV vaccination

1. How long have you been in this role?
2. Can you tell us about your role and responsibilities within this community regarding vaccination?
3. What knowledge/information do you have about the HPV vaccine?
4. What is your opinion on the HPV vaccine?

   Prompt(s): Benefit (or not) for the girl child and justify

5. What does your community think about the HPV vaccine?

   Prompt(s): Benefit (or not) for the girl child and justify

6. Has your community participated in activities related to HPV vaccination for young girls? *If so, what is your role in these activities? If not, why didn't she/he participate?*

7. How do you collaborate with other stakeholders in the implementation of HPV vaccination activities?

   Prompt(s): Emphasize collaboration with HFs, schools, CSOs, and other stakeholders

B. Knowledge and perceptions of the GNV approach

1. Did you know that the HPV vaccine is currently being administered to boys and girls in Cameroon?

   Prompt(s): Find out how he found out
2. What influence did the administration of the HPV vaccine to girls and boys have on the decision of members of your community to vaccinate their children?
3. Given the implementation of the GNV approach, what are the potential challenges that could limit its effectiveness in improving the uptake of the HPV vaccine in this community?
4. Are there specific factors that could facilitate the success of the GNV approach in this community? Why?

C. Linking Community Dialogue Strategies and Periodic Intensification of Routine Immunization (PIRIs)**to the Gender-Neutral Approach (COMMUNITY IMPLEMENTING PIRIs AND CD)**

1. What have been the impacts of the Periodic Intensification of Routine Immunization (PIRIs) and Community Dialogues (CDs) conducted in your community for HPV vaccine uptake?
   Relaunch: Name the most illustrative examples
2. What are the challenges encountered in the implementation of these activities that could affect the uptake of the HPV vaccine?
   Recovery: Challenges related to the ownership of these activities by health personnel
3. How were they overcome?
4. What is the impact of these activities on the perceptions and decisions of different stakeholders (religious leaders, parents, schools, etc.) regarding the vaccine in your community?
5. Are there any activities other than those mentioned above that have influenced the uptake of HPV vaccines? Please detail

D. Linking Community Dialogue Strategies and Periodic Intensification of Routine Immunization to the Gender-Neutral Approach (COMMUNITY THAT HAS NOT IMPLEMENTED PIRI AND CD)

1. What can you tell us about PIRIs and CDs in your community?
   Prompt: Insist on the PIRIs and CDs related to HPV
2. Do you think that combining these activities with the gender-neutral approach in your community can have a significant effect on the HPV vaccine uptake? Why?

E. Barriers and Facilitators

1. In your experience, what are the main reasons why some members of your community choose not to have their children vaccinated against HPV?
2. What factors do you think have encouraged others in your community to get their children vaccinated against HPV?
   Prompt(s): Provide examples of successful strategies or initiatives observed.

F. Recommendations

1. What recommendations can you make to improve the uptake of the HPV vaccine in the community?
2. What other things would you like to share with us about your experience and perspective on HPV vaccination and the GNV approach?

DO YOU HAVE ANYTHING ELSE TO ADD?
